# A Poluição por Partículas Finas é um Fator de Risco Importante para Doenças Cardiovasculares

**DOI:** 10.36660/abc.20260006

**Published:** 2026-02-26

**Authors:** Desiderio Favarato

**Affiliations:** 1 Universidade de São Paulo (HCFMUSP) Instituto do Coração Faculdade de Medicina São Paulo SP Brasil Instituto do Coração (Incor) do Hospital das Clínicas da Faculdade de Medicina da Universidade de São Paulo (HCFMUSP), São Paulo, SP – Brasil

**Keywords:** Poluição do Ar, Material Particulado, Doenças Cardiovasculares

Nesta edição, Moura et al.^1^ constataram que a região metropolitana do Rio de Janeiro, RJ, BR, apresenta níveis de poluição atmosférica extremamente elevados e que houve correlação direta entre a poluição atmosférica por MP_2,5_ (material particulado de 2,5 μm) e maior incidência de doenças cardiovasculares (DCV), especialmente em indivíduos de baixa condição socioeconômica e idosos.

Desde que o Estudo de Framingham enumerou os chamados fatores de risco tradicionais – hipertensão, colesterol LDL alto, colesterol HDL baixo, diabetes, tabagismo, obesidade, estilo de vida sedentário e sexo masculino – um número crescente de fatores de risco tem sido continuamente adicionado a essa lista.^2^

O conceito de aterosclerose como uma doença inflamatória crônica^1^ associou todos os fatores de risco ao seu início, crescimento e complicações, como síndrome coronariana crônica e aguda, acidente vascular cerebral e doença vascular periférica.

Tudo começa com a ativação ou inflamação endotelial, resultando na produção e exposição de moléculas de adesão (selectinas, integrinas), molécula de adesão intercelular-1 (ICAM-1) e molécula de adesão plaquetária-endotelial-1 (PECAM-1). Isso é seguido pela migração de monócitos e linfócitos para o espaço subintimal, promovendo a produção de citocinas e quimiocinas, fatores de crescimento e metaloproteinases da matriz (MMPs), além da migração de células musculares lisas vasculares. O processo culmina no crescimento da lesão e em complicações trombóticas por ruptura ou erosão.

Como a poluição do ar medida por MP_2,5_ é inserida neste cenário?

As MP (partículas sintéticas) são uma mistura complexa de partículas sólidas e matéria gasosa inaladas, com composição variável, desde fontes naturais, como incêndios florestais e tempestades de areia, até fontes antropogênicas, como a combustão de combustíveis fósseis, o transporte e os processos industriais.

As partículas mais perigosas são as de 2,5 micrômetros, pois podem atingir os pulmões, entrar na circulação sanguínea sistêmica e desencadear um processo inflamatório sistêmico.^3^

O relatório Global Burden of Disease 2019 colocou a poluição do ar na quarta posição de risco para mortalidade por todas as causas, superada apenas por hipertensão, tabagismo e riscos alimentares. Acima de hiperglicemia de jejum, alto índice de massa corporal, colesterol LDL elevado e disfunção renal.^4^

Uma metanálise de 11 estudos de coorte europeus mostrou um aumento de 13% nos eventos de síndrome coronariana aguda com um aumento de 5 μg/m^3^ na exposição média anual estimada a MP_2,5_ e um aumento de 12% nos eventos de síndrome coronariana aguda com um aumento de 10 μg/m^3^ na exposição média anual estimada a MP10.^5^

Mesmo a exposição de curto prazo a MP também está associada a uma maior incidência de infarto agudo do miocárdio (IAM), principalmente IAM com supradesnivelamento do segmento ST, e mortalidade relacionada, particularmente em pacientes idosos com doença arterial coronariana preexistente e fatores de risco significativos para DCV.^6^

Um estudo recente revelou que a razão de risco para todos os eventos cerebrovasculares foi 2,14 vezes maior ao comparar os quartis superior e inferior de MP_2,5_.^7^

Além de ser um fator de risco cardiovascular direto, o MP_2,5_ também é um marcador de outros poluentes atmosféricos, como ozônio, monóxido de carbono, óxidos de nitrogênio, óxidos de enxofre e chumbo. Todos estão relacionados à inflamação sistêmica, ao estresse oxidativo e à disfunção endotelial, podendo levar à aterosclerose.

Além do potencial aterosclerótico, as MP_2,5_ atuam sobre os fatores de risco tradicionais, aumentando seu poder aterogênico.

A qualidade do ar em ambientes internos e externos, incluindo o ar no local de trabalho, é um problema de saúde pública, e medidas rigorosas devem ser tomadas para seu controle, visando uma melhor assistência e contribuindo para uma abordagem mais completa da saúde.
