# Supplementary material for: A Poluição por Partículas Finas é um Fator de Risco Importante para Doenças Cardiovasculares
Source: Arq Bras Cardiol. 2026 Feb 26;123(1):e20260006. [Article in Portuguese] doi: 10.36660/abc.20260006 (PMC13128173; doi:10.36660/abc.20260006)
Supplement: Translation

# Translation: Fine Particulate Pollution is a Powerful Risk Factor for Cardiovascular Disease

**DOI:** 10.36660/abc.20260006i

**Authors:** Desiderio Favarato

**Affiliations:** 1 São Paulo SP Brazil Instituto do Coração (Incor) do Hospital das Clínicas da Faculdade de Medicina da Universidade de São Paulo (HCFMUSP), São Paulo, SP – Brazil

**Keywords:** Air Pollution, Particulate Matter, Cardiovascular Diseases

In this issue, Moura PH et al^1^found that the Rio de Janeiro Metropolitan area, RJ, BR presents out-of-limit high levels of air pollution and there was a direct correlation between air pollution with PM_2.5_(Particulate Matter of 2.5 μm) and higher incidence of cardiovascular disease, especially in lower socioeconomic status and in the elderly.

Since the Framingham Study had enumerated the so-called traditional risk factors – hypertension, high LDL-cholesterol, low HDL-cholesterol, diabetes, tobacco smoking, obesity, sedentary lifestyle, and male sex – a growing number of risk factors have been continuously added to that list.^2^

The concept of atherosclerosis as a chronic inflammatory disease^1^has linked all risk factors to its initiation, growth, and complications such as chronic and acute coronary syndrome, stroke, and peripheral vascular disease.

All begins with endothelial activation or inflammation, resulting in the production and exposure of adhesion molecules (selectins, integrins), Intercellular Adhesion Molecule-1 (ICAM-1), and Platelet Endothelial Cell Adhesion Molecule-1 (PECAM-1). That is followed by monocytes and lymphocytes migration to the sub-intimal space, promoting production of cytokines and chemokines, growth factors, and matrix metalloproteinases (MMP), and migration of vascular smooth muscle cells. Ending in lesion growth and thrombotic complications by rupture or erosion.

How is air pollution measured by PM_2.5_inserted in this scenario?

PM is a complex mixture of inhaled solid particles and gaseous matter, with a variable composition from natural sources: wildfires and sand dust storms, to anthropogenic sources: combustion of fossil fuel, transportation, and industrial processes.

The worst PM are of 2.5 micrometers because they can reach to lung and enter the systemic blood circulation and elicits systemic inflammation process.^3^

The Global Burden of Disease 2019 report has shown air pollution in the fourth position of risk of all-cause mortality, only overcome by hypertension, tobacco, and dietary risk. Above high fasting plasma glucose, high body-mass index, high LDL-cholesterol, and kidney dysfunction.^4^

A meta-analysis of 11 European cohort studies showed a 13% increase in acute coronary syndrome events with a 5 μg/m^3^increase in the estimated annual mean PM_2.5_exposure and a 12% increase in acute coronary syndrome events with a10 μg/m^3^increase in the estimated annual mean PM10.^5^

Even short-term exposure to PM is also linked to an increased incidence of acute myocardial infarction (AMI), mainly ST-segment elevation AMI, and related mortality, particularly in elderly patients with preexisting CAD and significant risk factors for CVD.^6^

A recent study found that the HR for all cerebrovascular events was 2.14 times higher when comparing the top vs bottom quartiles of PM_2.5_.^7^

Besides being a direct cardiovascular risk factor, PM_2.5_is also a marker of other air pollutants such as ozone, carbon monoxide, nitrogen oxides, sulfur oxides, and lead. All are also related to systemic inflammation, oxidative stress, and endothelial dysfunction, and eventually atherosclerosis.

In addition to atherosclerotic potential, PM_2.5_acts on traditional risk factors, increasing their atherogenic power.

Living air environment, both indoor and outdoor, and the workplace, is a public health problem, and major enforcement has to be made for its control for better care, which may contribute to a more complete approach to health care.
